# Identifying Change Points in a Covariate Effect on Time-to-Event Analysis with Reduced Isotonic Regression

**DOI:** 10.1371/journal.pone.0113948

**Published:** 2014-12-04

**Authors:** Yong Ma, Yinglei Lai, John M. Lachin

**Affiliations:** 1 The Biostatistics Center, George Washington University, Rockville, MD, United States of America; 2 Department of Epidemiology and Biostatistics, Milken Institute School of Public Health, George Washington University, Washington, DC, United States of America; 3 Department of Statistics, George Washington University, Washington, DC, United States of America; Yale School of Public Health, United States of America

## Abstract

Isotonic regression is a useful tool to investigate the relationship between a quantitative covariate and a time-to-event outcome. The resulting non-parametric model is a monotonic step function of a covariate *X* and the steps can be viewed as change points in the underlying hazard function. However, when there are too many steps, over-fitting can occur and further reduction is desirable. We propose a reduced isotonic regression approach to allow combination of small neighboring steps that are not statistically significantly different. In this approach, a second stage, the reduction stage, is integrated into the usual monotonic step building algorithm by comparing the adjacent steps using appropriate statistical testing. This is achieved through a modified dynamic programming algorithm. We implemented the approach with the simple exponential distribution and then its extension, the Weibull distribution. Simulation studies are used to investigate the properties of the resulting isotonic functions. We apply this methodology to the Diabetes Control and Complication Trial (DCCT) data set to identify potential change points in the association between HbA1c and the risk of severe hypoglycemia.

## Introduction

In clinical practice, disease diagnosis and subsequent treatment are often guided by a strict threshold (i.e. change point) of a biomarker. For example, fasting plasma glucose (FPG) at 126 mg/dl is the cutoff to diagnose type II diabetes, and more intensive treatment is used when FPG reaches 140 mg/dl. Such change points are often identified through a large scale health study where disease risk increases substantially when a biomarker level exceeds a change point. Because identifying change points is data driven, more recent research data would mandate the update of the change points. In the case of diabetes diagnosis, the diagnostic threshold was at FPG ≥140 mg/dl before 1997. However, in 1997, increased cardiovascular and micro-vascular disease risk at lower values prompted the American Diabetes Association to recommend lowering the diagnostic threshold to 126 mg/dl. Changes like this have huge effects on medical practice, especially the initiation of a treatment, hence a systematic approach to identify change points in a covariate is well worth the effort.

Ancukiewicz et al. [1] have established an isotonic regression method to model the relationship between a quantitative covariate and clinical events. The covariate is assumed to be discrete with multiple levels so that the model provides an estimate of the outcome at every discrete value of the covariate. The resulting model is a step function where each new step can be viewed as a change point. They used their method to identify a change point in the association of CD4 count with HIV risk and the method worked well. However, in situations where the data is dense, that is, there is a large number of subjects with the outcome event and support over many discrete levels of the covariate, the model can also include many mini-steps and further combination of some mini-steps is desirable. Schell and Singh (1997) [2] proposed the idea of ‘reduced isotonic regression’ in which a backward elimination procedure is used after the usual isotonic regression model is built. Salanti and Ulm (2005) [3] also proposed a two-step procedure to estimate threshold limit values with binary outcomes. In their approach, the second stage in the algorithm is a sequence of Fisher tests for the adjacent 2×2 tables to accomplish a reduced model. Very recently, Han et al. (2013) [4] proposed to use a reduced piecewise exponential approach to improve the modeling of survival time. They also used a two step procedure in which all insignificant change-points are eliminated after first implementing an order restriction on the failure rate. A flaw in the two stage approach is that the resulting model may not give the global maximum likelihood. Thus, we propose to employ a global optimization approach, examining all potential combinations of isotonic models with the constraint that the adjacent steps are significantly different and then identify the one with the maximum likelihood. We implemented this approach with a modified dynamic programming algorithm proposed by Lai [5]. This approach was chosen over the popular *pool adjacent violators algorithm* (PAVA) because the later cannot guarantee a global optimization solution when the extra testing is required. Lai and Albert [6] described using the approach in a linear mixed effects model, here we apply the approach in a parametric time-to-event data analysis.

In a nutshell, the algorithm examines all observed covariate 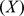 values, from the smallest (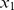) to the largest (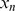), one at a time. At each 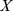 value, the algorithm will partition the values smaller or equal to 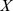 and identify an optimal step function satisfying the following three criteria: the function is isotonic, the distributions between two adjacent steps are significantly different, and the optimal step function has the maximum likelihood among all possible step functions that meet the first two criteria. In the process of finding the optimal partition, all the other partitions that satisfy the first two conditions are recorded and saved for future use. This unique feature reduces the computing time from the 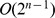 in a naive try to 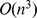, assuming that there are 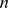 possible 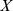 values. The detailed description and related mathematical proofs about the modified dynamic programming algorithm was published elsewhere [5].

Large scale clinical trials like the Diabetes Control and Complication Trial (DCCT) [7] and the UK Prospective Diabetes Study (UKPDS) [8] demonstrated that improved glycemic control, represented by HbA1c (approximately a function of the 12 week average of glucose), reduces microvascular complications. However, they also showed that a lower glucose level is associated with elevated risk of severe hypoglycemia. It is therefore critical to identify the change points in the association between HbA1c and hypoglycemia to help establish a glycemic target which is low enough to minimize microvascular risk and yet not so low as to increase the risk of severe hypoglycemia. We apply this methodology to the DCCT data set to identify such change points.

## Methods

As in a parametric regression approach for time-to-event data, the null hypothesis here is that the covariate of interest 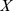 is not associated with survival time. The alternative hypothesis is that there exists at least one 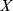 value where the survival function changes significantly after reaching this value. If there are more than one change point, the change in survival function is monotonic. Without loss of generality, we only present the monotonically increasing scenario. Assuming that 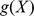 is a parameter in the survival time distribution, the hypothesis testing can be described as the following
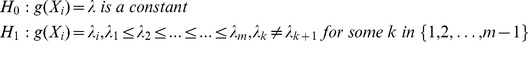
(1)


To establish a reduced isotonic regression as proposed, we need to specify the underlining survival function first. We start with the simple exponential distribution with a constant hazard in terms of time and then extend the results to the more robust Weibull distribution.

### Survival Time with an Exponential Distribution

When the event times follow an exponential distribution with constant hazard rate 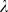, the survival function can be expressed as 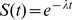
(2)where 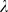 can be expressed as an isotonic step function of the covariate 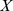. The goal of the algorithm is to determine whether each 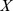 value can be combined with its neighbors so that the final step function meets the three criteria described previously: 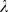 being monotonically increasing, adjacent steps being statistically significantly different and having the overall maximum likelihood. The algorithm starts at the smallest covariate value 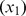 and moves on until the final optimization is achieved.

Assume that 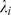 is the hazard associated with the 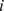
th level of 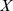 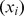 where there are 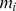 observations. The time-to-event data for the 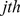 participant in this group is represented as 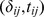, where 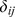 is the censoring indicator (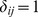 indicating an event or 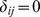 indicating right-censoring) and 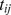 is the survival time.

The log likelihood for all 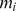 observations can be expressed as
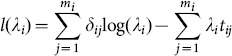
(3)and 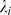 can be estimated as
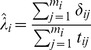
(4)


Now that the 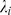 is estimated, we want to compare this 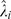 with the next one 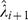 so that the estimates are monotonically increasing and significantly different. We use Cox's *F*-test for the statistical testing since it is the most powerful test for comparing two exponential distributions [9] [10]. Assuming 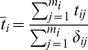
(5)


The ratio of the two 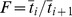 follows a *F*-distribution with 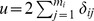 and 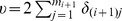 degrees of freedom. The test reject 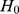 if 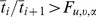 where the nominal 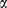 is pre-specified. When either 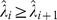 or the 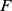 test is not significant at 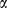, the two 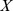 steps are combined and treated as a single step and the procedure continues. There are usually multiple partitions that will satisfy both the isotonic and significance criteria, among them, the one with the largest likelihood function is chosen as the optimization. The detailed algorithm used in the optimization is presented in the Supporting Information section.

### Survival Time with a Weibull Distribution

The Weibull distribution is an extension of the exponential distribution and its shape parameter, 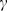, determines the shape of the distribution of survival times. The Weibull survival function can be expressed as 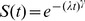
(6)


It is well known that, if a random variable 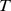 follows a Weibull distribution with parameters 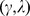, then 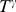 will follow an exponential distribution with parameter 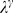. Therefore, for a given 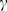, a simple power transformation of the survival times yields an exponential distribution. With this feature we can obtain estimates under a Weibull assumption by employing the algorithm already developed for the exponential distribution with power-transformed data. We assume that 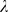 is a step function of 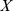, and 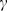 is a constant which will be estimated together with 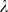.

The log likelihood function for the observations with covariate value 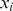 under the Weibull assumption is
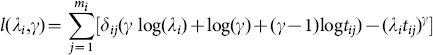
(7)and the likelihood for all data is
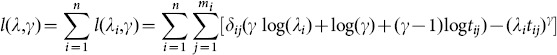
(8)


We use the following iterative steps to estimate 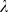 and 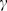.

Step 1: Estimate 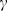 by assuming that all observations are independently identically distributed (*i.i.d.*) from the same Weibull distribution with parameters 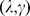, i.e., 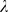 is the same for all 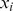.

Step 2: With 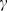 estimated, we transform the survival time 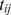 to 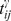 and use the algorithm developed in the exponential case to estimate 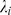


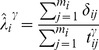
(9)


Step 3: We update 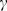 with a MLE estimator by solving the following equation derived from (8)
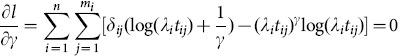
(10)


Step 4: Repeat steps 2 and 3 until the 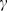 estimate converges, which is defined as change in 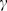 is less than 0.1%.

Both the exponential and the Weibull algorithms have been implemented in the R statistical system and the codes can be found in the supplemental material.

## Results

### An Example

**Figure 1:**
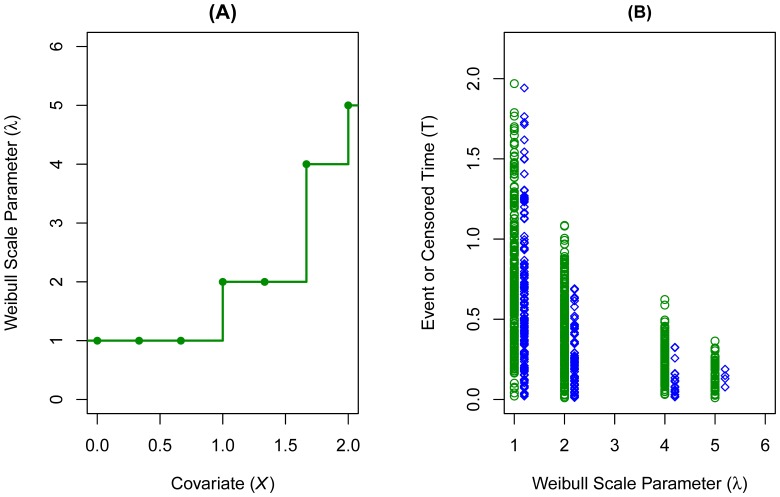
Underlying true model and time-to-event data. (A) Weibull scale parameter 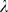 is an isotonic function of 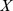. (B) Event or censored time follows the Weibull distribution with a fixed shape parameter (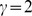) and scale parameter 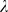 shown in (A). The green open circles represent event times and the blue open diamonds represent censored times.

We illustrate the algorithm using the following hypothetical example depicted in Figure 1. We simulated time-to-event data that follows a Weibull distribution. 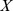 was the covariate of interest and had 7 distinct values 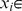 {0, 1/3, 2/3, 1, 4/3, 5/3, 2}, evenly spaced. A data set of 1000 observations was then generated by sampling a value of 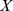 from the set where the extremes each had probability 1/12*th* and the other 5 values had probability 1/6*th*. The corresponding hazard rates 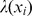 were determined by the step function shown in Figure 1 (A) and the shape parameter 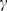 was set at 2.

To generate the event time T, we used the known fact that if 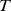 followed a Weibull distribution with parameters 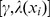 then 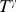 would follow an exponential distribution with patameter 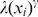. We randomly generated an event time 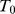 from the exponential distribution with rate 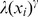. Thus, a back transformation of 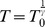 would create a random variable 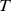 following a Weibull distribution 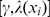. For censoring, we used independently generated random numbers following uniform distributions in the interval between the minimum and the maximum of the event times as the censoring time 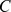. The minimal of the event time and the censoring time 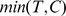 was used as the final survival time. The event indicator 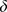 was coded as 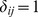 when 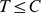 or 0 otherwise. Figure 1 (B) displays survival times with open circles representing event times (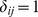) and open diamonds representing censored times (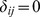).

Table 1 shows the distinct values of 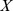, number of subjects with each value of 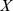, true hazard rates 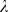 and the initial individually estimated hazard rates and their standard errors (with 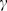 estimated at 2.02 in the final iteration of the algorithm). Before the constraint of monotonicity, the seven distinct estimates (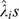) were close to the true values (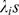), however, no change point(s) could be determined because each 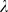 is a distinct value.

**Table 1:** Sample data following Weibull distributions with 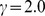 and 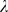 as a step function of 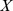.

True Parameters							
Steps	1			2		3	4
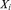	0	1/3	2/3	1	4/3	5/3	2
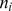	94	159	166	170	160	184	67
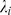	1	1	1	2	2	4	5
**Initial Estimates**							
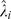	1.09	1.04	0.95	2.02	1.91	3.87	5.31
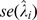	0.07	0.05	0.05	0.09	0.08	0.15	0.33

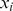
: covariate with 7 distinct values; 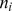: number of observations at each 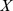 value; 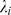: step function of 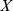 with 4 distinct values; 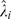: 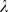 estimated at each 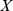 value before the implementation of the reduced isotonic regression algorithm; 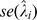: standard error of the 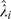.

**Figure 2:**
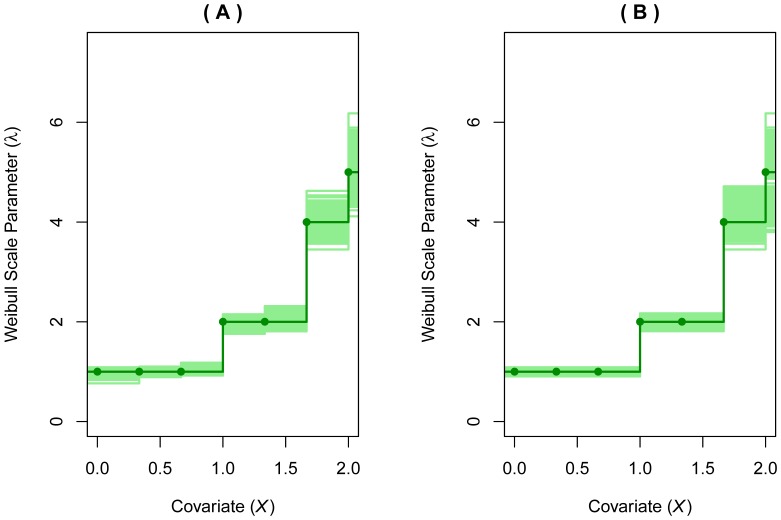
Simulation results from 1000 repetitions. (A) Regular isotonic regression without testing between steps. (B) Reduced isotonic regression with nominal 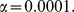 The dark green lines represent the underlying true model.

We applied the algorithm to the data set to obtain a reduced isotonic regression model. The same example was repeated 1000 times, each time with a slightly different random data set and the results are shown in Figure 2. Panels (A) corresponds to models from the regular isotonic regression and panel (B) from the reduced isotonic regression with pre-specified testing significance at 0.0001. In panel (B), the bands of the estimates around the true 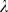 values at 1 and 2 are much tighter, indicating improved model fit from incorporating the significance testing. The very small nominal 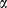 was chosen for this example to demonstrate the effect of statistical testing. Such a stringent significance level could be too strict for real world data and shouldn't always be used.

### Small Sample Performance

A good modeling strategy is a strategy that still works when sample size is small. In the case of time-to-event data, due to censoring, the statistical information depends on the number of subjects experiencing the event, which is smaller than the number of participants in the study. Here we evaluate the performance of the reduced isotonic regression employing combinations of sample size and percent of censoring that yields 500, 200, 100 and 50 events.

We used the previously described example again. Adjacent steps were tested at nominal 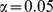 and each scenario was repeated 1000 times. The results are shown in Table 2 and Table 3. Table 2 describes the frequency of various steps we identify in the 1000 repetitions. Table 3 summarizes the means and mean squared errors of the estimates of 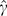 and 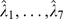. As the number of events decreases, the number of correctly identified steps (4) decreases and the model estimates are more likely to be biased with bigger variances. However even when the number of events is as small as 50, the models are able to capture the pattern of the underlining true model at about 25% of the times and only miss 1 step at 70% of the times. The parameter estimates are reasonably close to the true parameters.

**Table 2:** Number of steps identified with various event numbers and percent censored.

Event N	Sample N	% Censored mean(range)	Number of Steps				
			2	3	4	5	6
∼500	1000	50.5(37.4–60.1)	0	86	785	126	3
	800	37.9(26.4–47.4)	0	126	745	122	7
	500	0	0	223	680	96	1
∼200	400	50.7(34.3–62.5)	0	376	527	95	2
	320	37.5(24.1–49.1)	0	440	485	72	3
	200	0	0	509	429	59	3
∼100	200	50.8(30.5–65.5)	0	550	384	62	4
	160	37.4(23.1–53.1)	0	577	372	49	2
	100	0	0	621	326	51	2
∼50	100	50.5(31.0–70.0)	20	694	266	20	0
	80	37.4(20.0–60.0)	8	699	268	25	0
	50	0	19	737	229	18	0

The combination of “Sample N” and “%censore” is used to yield the targeted number of events in the “Event N” column, repeated 1000 times.

**Table 3:** Precision of parameter estimates with various event numbers and percent censored.

Event N	Sample N	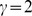	x =	0	1/3	2/3	1	4/3	5/3	2
			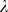 =	1	1	1	2	2	4	5
mean and mean squared error for the estimates of 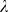										
∼500	1000	2.01		0.99	1.00	1.01	1.99	2.01	4.02	5.00
		0.0051		0.0043	0.0027	0.0030	0.0061	0.0065	0.040	0.13
	800	2.01		0.99	1.00	1.01	1.99	2.01	4.02	4.99
		0.0052		0.0037	0.0022	0.0025	0.0072	0.0073	0.044	0.16
	500	2.01		0.99	1.00	1.01	1.99	2.01	4.06	4.95
		0.0050		0.0021	0.0014	0.0017	0.0079	0.0085	0.072	0.22
∼200	400	2.01		0.97	1.00	1.01	2.00	2.01	4.10	4.90
		0.014		0.013	0.0081	0.0093	0.015	0.016	0.11	0.39
	320	2.02		0.99	1.00	1.01	1.99	2.02	4.10	4.85
		0.013		0.0078	0.0053	0.0062	0.016	0.018	0.12	0.47
	200	2.03		0.99	1.00	1.01	2.00	2.02	4.14	4.86
		0.015		0.0047	0.0035	0.0055	0.018	0.022	0.18	0.59
∼100	200	2.04		0.97	1.01	1.03	1.99	2.04	4.16	4.83
		0.030		0.023	0.016	0.022	0.039	0.043	0.22	0.66
	160	2.05		0.98	1.01	1.03	1.99	2.04	4.20	4.82
		0.030		0.019	0.012	0.016	0.034	0.042	0.27	0.65
	100	2.06		0.98	1.00	1.03	2.01	2.05	4.18	4.89
		0.032		0.0088	0.0070	0.014	0.041	0.055	0.32	1.28
∼50	100	2.07		0.97	1.00	1.08	1.96	2.07	4.17	4.82
		0.069		0.043	0.044	0.085	0.11	0.13	0.35	1.36
	80	2.08		0.97	1.02	1.07	1.98	2.10	4.23	4.90
		0.072		0.032	0.027	0.057	0.11	0.14	0.53	1.65
	50	2.14		0.99	1.02	1.07	1.98	2.13	4.24	4.99
		0.091		0.021	0.020	0.050	0.11	0.24	0.79	2.60

For the mean and mean squared error columns, the first row is the mean and the second row is the mean squared error. Percent and range of censoring is the same as shown in Table 2.

### Model Diagnostics and Other Features

Cox-Snell [11] residuals can be applied to assess whether the model assumptions are accurate. If the model fits the data, and we plot Cox-Snell residual 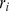 against the negative log of the survival function of the residual 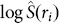, it should be a straight line with unit slope and zero intercept.

Although the nominal significance between the steps of the final model is pre-specified (herein at level 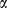), such testing between any two steps does not provide an overall test of the significance of the covariate effect in the reduced isotonic regression model. Under certain conditions the likelihood ratio test of the covariate significance may follow a chi-square distribution. However, the degrees of freedom is unknown. We propose to use a permutational approach to obtain the distribution of the likelihood ratio test under the null. This distribution will allow us to calculate the p-value of the covariate of interest.

It is still difficult to understand the theoretical properties of the parameters estimated from the reduced isotonic regression algorithm. To circumvent the problem, we use the distribution free bootstrap approach [12] to calculate the confidence intervals of the parameter estimates. A bootstrap sample with replacement is created from the original data set and model parameters are generated. This is repeated multiple times and a distribution of the parameter estimates is created. The 95% confidence intervals for the parameter estimates is therefore constructed.

### Application

The Diabetes Control and Complication Trial (DCCT) was a clinical trial aimed at comparing intensive treatment, i.e., at least 3 insulin injections a day, to the traditional treatment, once or twice a day for Type 1 diabetes mellitus (T1DM) patients. Although the intensive treatment significantly delayed the onset and slowed the progression of retinopathy, neuropathy and nephropathy, there is a two-to-three fold increase in episodes of severe hypoglycemia (low blood sugar) that could lead to coma (unconsciousness) and/or siezures [7]. Here we use the methods developed in the previous sections to explore the relationship between HbA1c and severe hypoglycemia for the 711 participants in the intensive treatment group. The DCCT hypoglycemia data is described by Lachin [13] and can be obtained from the following web site: http://www2.bsc.gwu.edu/bsc/webpage.php?no=18.

**Figure 3:**
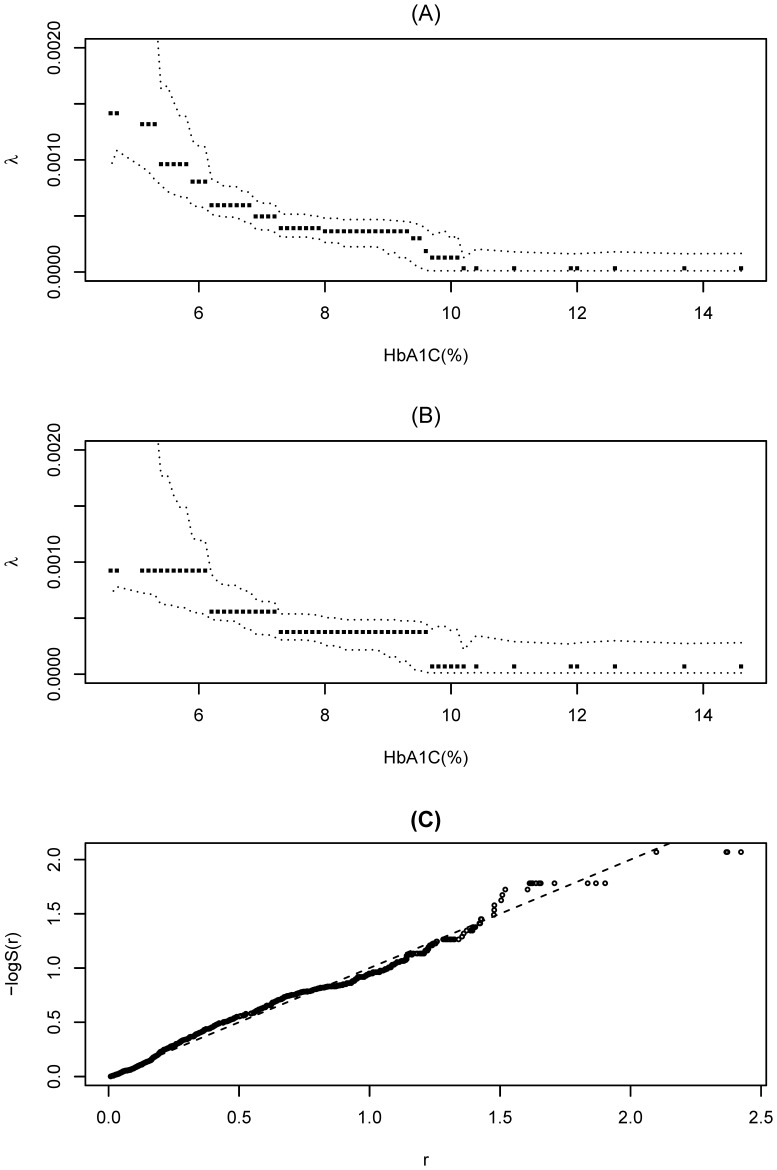
Modeling HbA1c and risk of severe hypoglycemia. (A) Regular isotonic regression without testing between steps. (B) Reduced isotonic regression with nominal 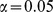 (C) Cox-Snell residual plot of Model B. Dotted lines in (A) and (B) represent 95% Confidence Intervals of 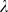.

The event is the first occurrence of severe hypoglycemia and the covariate of interest is the participant's HbA1c at study entry. We applied the reduced isotonic regression with Weibull assumption and a nominal 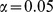 to the data and present the results in Figure 3. Panel (A) is the regular isotonic regression without testing between steps and the resulting model has many small steps. When the testing between steps is added, a parsimonious model with only 3 change points (6.2, 7.3 and 9.6) is obtained. Model in panel (B) suggests that even though it is ideal to lower patients' HbA1C level to as close to normal (5.6 or lower) as possible, we need to monitor the level closely when it crosses 9.6, 7.3, and 6.2 to avoid the occurrence of severe hypoglycemia. Overall significance of HbA1C is 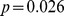, estimated from the permutation approach. The model estimate for the shape parameter is 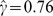 (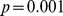), suggesting that the hypoglycemic events tend to occur early in the implementation of the intensive therapy. The Cox-Snell residual plot (panel (C)) indicates that the Weibull assumption is valid.

## Discussion

Herein we demonstrated how reduced isotonic regression can be implemented in parametric time-to-event data analysis with survival time following an exponential or Weibull distribution.

As shown in the simulation studies, adding statistical testing between steps can reduce number of steps falsely introduced by noise. Although in the simulation example we chose 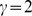 for clarity of presentation, we have examined the scenario when 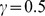 and obtained similar results [14]. A 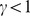 might be more representative of medical data of chronic diseases and the fact that our approach worked well with the DCCT data (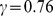) is reassuring. In the DCCT example, the regular isotonic regression model produced too many change points and were not directly useful for the clinical practice. Although we could identify a couple change points from the regular model by eyeballing the figure, it is not systematic and very subjective. By using the statistical testing we were able to build a parsimonious model with only a few change points. Obviously, the nominal isotonic testing level 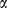 will influence the number of change points. As the nominal 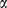 becomes smaller the number of change points decreases. As the methodology allows for user's choices of the nominal 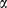, in real world data analysis, we recommend to start with a big 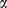 at 1.0, i.e., no testing done between steps, to obtain an exploratory check of the association between the covariate and the outcome. After that, a smaller nominal 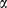 can be applied to obtain a more parsimonious model with fewer change points for practical use. A methodological approach such as those used in choosing the smoothing parameter value in non-parametric data analysis can be developed to choose a single best nominal 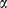, however, it is beyond the scope of this paper with both a caveat and possible extension of the method given.

In health research or epidemiological studies, we often want to evaluate whether a covariate of interest is associated with the outcome independently of the effects of other covariates. This is usually achieved by adding (or adjusting for) other covariates known to be associated with the outcome in the model. In this case, we can add the known covariates to the algorithm and solve for them simultaneously with the covariate of interest. Estimates of these covariates can be solved in the same way as the shape parameter 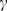 in the Weibull case, i.e., held as constants while solving for the parameters related to 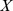.

## Supporting Information

Appendix S1 **The modified dynamic programming algorithm.**
(PDF)

Appendix S2 **R programs developed for the reduced isotonic regression in survival analysis.**
(TXT)
