# Supplementary material for: Identifying Change Points in a Covariate Effect on Time-to-Event Analysis with Reduced Isotonic Regression
Source: PLoS One. 2014 Dec 4;9(12):e113948. doi: 10.1371/journal.pone.0113948 (PMC4256386; doi:10.1371/journal.pone.0113948)
Supplement: Appendix S1 — The modified dynamic programming algorithm. (PDF) [file pone.0113948.s001.pdf]

# Appendix S1

## The modified dynamic programming algorithm

A detailed flow chart describing the iterative process has already been published (See Lai 2011 DOI: 10.1371/journal.pone.0019754, Figure 1). Here we give a brief description of the process involving the exponential distribution. A set $V_i$ containing three elements (link, index, loglik) is built at each $X$ level. The link is a counter that associates the current $X$ under evaluation to the ones already evaluated, e.g., the ones smaller than $X$ when $X$ is increasing. The index records the ones meeting all three criteria among the links examined. The loglik is the log of likelihood of all the data up to the $X$ value under evaluation.

$link[1] \leftarrow 1; index[1] \leftarrow 0; \hat{\lambda}_1 \leftarrow \frac{1}{t_1}; loglik[1] \leftarrow \log \hat{\lambda}_1 - \hat{\lambda}_1 t_1;$
$V[1] \leftarrow (link[1], index[1], loglik[1])$
**for i=2 to n do {**
$link[i] \leftarrow 1; \quad index[i] \leftarrow 0; \quad \hat{\lambda}_i \leftarrow \frac{i}{\sum_{j=1}^{i} t_j};$
$loglik[i] \leftarrow i \log \hat{\lambda}_i - \hat{\lambda}_i \sum_{j=1}^{i} t_j;$
$V[i] \leftarrow (link[i], index[i], loglik[i])$
**for j=1 to i-1 do {**
**for k=1 to length of all the links in V[j] do till a link is found{**
$link \leftarrow$ kth link associated with V[j]
$\hat{\lambda}_1 \leftarrow \frac{i-j}{\sum_{p=j+1}^{i} t_p}; \quad \hat{\lambda}_0 \leftarrow \frac{j-link+1}{\sum_{p=link}^{j} t_p};$
**if** $\hat{\lambda}_1 \geq \hat{\lambda}_0$ **and** $\hat{\lambda}_1 \neq \hat{\lambda}_0$ **is significant at** $\alpha$ **then do {**
$link \leftarrow j; \qquad index \leftarrow k$
$loglik \leftarrow loglik[S_j]_k + [i-j] \log \hat{\lambda}_1 - \hat{\lambda}_1 \sum_{p=j+1}^{i} t_p$
include $(link, rank, loglik)$ as a new element in $V_i$
**}**
**}**
**}**
Sort $V_i$ according to the decreasing order of loglik
**}**
